# Patient and provider determinants of nephrology referral in older adults with severe chronic kidney disease: a survey of provider decision making

**DOI:** 10.1186/1471-2369-12-47

**Published:** 2011-09-26

**Authors:** Kellie H Campbell, Sandy G Smith, Joshua Hemmerich, Nicole Stankus, Chester Fox, James W Mold, Ann M O'Hare, Marshall H Chin, William Dale

**Affiliations:** 1 University of Chicago, Section of Geriatrics and Palliative Medicine, 5841 South Maryland Avenue, MC 6098, Chicago, Illinois 60637, USA; 2 University of Chicago, Pritzker School of Medicine, 924 East 57 th Street, BSLC 013A, Chicago, Illinois 60637, USA; 3 University of Chicago, Section of Nephrology, 5841 South Maryland Avenue, MC 5000, Chicago, Illinois 60637, USA; 4 University of New York at Buffalo, Department of Family Medicine, 462 Grider Street, Buffalo, New York, 14215, USA; 5 University of Oklahoma, Department of Family Medicine, 900 North East 10 th Street, Oklahoma City, Oklahoma, 73104, USA; 6 VA/Puget Sound Medical Center, 1660 South Columbian Way, Seattle, Washington, 98108, USA; 7 University of Chicago, Section of General Internal Medicine, 5841 South Maryland Avenue, MC 2007, Chicago, Illinois, 60637, USA

## Abstract

**Background:**

Although chronic kidney disease (CKD) disproportionately affects older adults, they are less likely to be referred to a nephrologist. Factors that influence the referral decisions of primary care providers (PCPs) specifically for older CKD patients have been incompletely described. Patient factors such as dementia, functional disability, and co-morbidity may complicate the decision to refer an older adult. This study evaluated the role of patient and PCP factors in the referral decisions for older adults with stage 4 CKD.

**Methods:**

We administered a two-part survey to study the decisions of practicing PCPs. First, using a blocked factorial design, vignettes systematically varied 6 patient characteristics: age, race, gender, co-morbidity, functional status, and cognitive status. CKD severity, patient preferences, and degree of anemia were held constant. Second, covariates from a standard questionnaire included PCP estimates of life expectancy, demographics, reaction to clinical uncertainty, and risk aversion. The main outcome was the decision to refer to the nephrologist. Random effects logistic regression models tested independent associations of predictor variables with the referral decision.

**Results:**

More than half (62.5%) of all PCP decisions (n = 680) were to refer to a nephrologist. Vignette-based factors that independently decreased referral included older patient age (OR = 0.27; 95% CI, 0.15 to 0.48) and having moderate dementia (OR = 0.14; 95%CI, 0.07 to 0.25). There were no associations between co-morbidity or impaired functional activity with the referral decision. Survey-based PCP factors that significantly increased the referral likelihood include female gender (OR = 7.75; 95%CI, 2.07 to 28.93), non-white race (OR = 30.29; 95%CI, 1.30 to 703.73), those who expect nephrologists to discuss goals of care (OR = 53.13; 95%CI, 2.42 to 1168.00), those with higher levels of anxiety about uncertainty (OR = 1.28; 95%CI, 1.04 to 1.57), and those with greater risk aversion (OR = 3.39; 95%CI, 1.02 to 11.24).

**Conclusions:**

In this decision making study using hypothetical clinical vignettes, we found that the PCP decision to refer older patients with severe CKD to a nephrologist reflects a complex interplay between patient and provider factors. Age, dementia, and several provider characteristics weighed more heavily than co-morbidity and functional status in PCP referral decisions. These results suggest that practice guidelines should develop a more nuanced approach to the referral of older adults with CKD.

## Background

Chronic kidney disease (CKD) is a recognized public health concern that preferentially affects older adults, more than a third of whom over age 70 meet the formal definition of CKD [1,2]. According to current Kidney Disease Outcomes Quality Initiative (KDOQI) guidelines, patients with severe CKD (a glomerular filtration rate (GFR) of less than 30 ml/min/1.73 m^2) ^should be referred to a nephrologist to manage the potential complications of CKD, slow the progression to end stage kidney disease, prepare patients for renal replacement therapy, and reduce mortality [3-5]. However, older adults though more likely to have severe CKD, are less likely to be referred to a nephrologist [5-7]. Previous studies of both primary care (PCPs) and nephrology providers suggest that more severe kidney disease, increased complexity of medical co-morbidity, limited life expectancy and patient preferences against dialysis, in addition to older age, influence provider referral decisions [8-11]. Our own work suggests that providers also consider the cognitive and functional status of older patients when deciding whether or not to refer the patient to a nephrologist [12]. In addition to patient factors, PCP characteristics might explain some of the variability in referral decisions [11,13]. However, precisely how PCPs prioritize and incorporate these patient factors into their decision-making strategy when addressing individual patients is not known. We previously outlined a conceptual framework that describes the complexity of PCP referral decisions for CKD and incorporates three main components relevant to the referral decision: patient characteristics, provider characteristics, and disease characteristics [14,15]. This study was designed to systematically assess the role of specific patient characteristics while accounting for characteristics of practicing PCPs that might influence referral decisions for older adults with severe CKD.

## Methods

### Study Design

We developed an online survey with two components: 1) a series of hypothetical clinical vignettes specifically designed to evaluate the independent effect of selected patient factors on the PCP decision to refer older patients with CKD and 2) a survey questionnaire to evaluate provider and practice characteristics, reaction to clinical uncertainty, and risk aversion.

The clinical vignettes were randomly assigned by using a blocked factorial design which varied 6 patient characteristics across each vignette: age, race, gender, medical co-morbidity, functional status, and cognitive status. These factors were chosen based on prior work that indicated that the presence of geriatric co-morbidities might be important in provider decisions to refer older adults with advanced CKD [12]. Each patient characteristic was defined as follows: 1) age as either "young old" age 67 or "old old" age 80; 2) gender as male or female; 3) race as black or white; 4) co-morbidity as the presence or absence of New York Heart Association class III congestive heart failure [16]; 5) functional status as ambulation with a walker or without assistive device; and 6) cognitive status as the presence or absence of moderate dementia [17].

The blocked factorial design allows for identification of the independent associations for each individual patient factor (as well as for 2-factor interactions) by dichotomously varying each patient factor creating a total of 64 (2^6^) unique vignettes. The blocked factorial design limits respondent burden by reducing the number of vignettes each PCP received while retaining the randomized assignment of 6 selected patient characteristics [18]. Each provider answered a randomly assigned block of 8 vignettes [18] (Table 1).

**Table 1:** Example of random block of clinical vignettes provided to PCP respondents

Vignette 1	**67 **year old **white male **with history of diabetes, hypertension, and **NYHA class III heart failure **has chronic kidney disease with a GFR of 20 for greater than 3 months. He is ambulatory **without assistive device**. He has **no history of dementia**.
Vignette 2	**80 **year old **black male **with history of diabetes and hypertension has chronic kidney disease with a GFR of 20 for greater than 3 months. He is ambulatory **without assistive devices**. He has **no history of dementia**.

Vignette 3	**67 **year old **white female **with history of diabetes and hypertension has chronic kidney disease with a GFR of 20 for greater than 3 months. She is ambulatory but **requires use of a walker**. She has **no history of dementia**.

Vignette 4	**80 **year old **black female **with history of diabetes, hypertension, and **NYHA class III heart failure **has chronic kidney disease with a GFR of 20 for greater than 3 months. She is ambulatory but **requires use of a walker**. She has **no history of dementia**.

Vignette 5	**80 **year old **white female **with history of diabetes, hypertension, and **NYHA class III heart failure **has chronic kidney disease with a GFR of 20 for greater than 3 months. She is ambulatory **without assistive device**. She has **history of moderate dementia (MMSE 15)**.

Vignette 6	**67 **year old **black male **with history of diabetes, hypertension, and **NYHA class III heart failure **has chronic kidney disease with a GFR of 20 for greater than 3 months. He is ambulatory but **requires use of a walker**. He has **history of moderate dementia (MMSE 15)**.

Vignette 7	**67 **year old **black female **with history of diabetes and hypertension has chronic kidney disease with a GFR of 20 for greater than 3 months. She is ambulatory **without assistive device**. She has **history of moderate dementia (MMSE 15)**.

Vignette 8	**80 **year old **white male **with history of diabetes and hypertension has chronic kidney disease with a GFR of 20 for greater than 3 months. He is ambulatory but **requires use of a walker**. He has **history of moderate dementia (MMSE 15)**.

Bolded items represent patient characteristics that were systematically varied.

We held other important patient factors constant for each vignette. Patients were described as having no strong preferences about referral and would defer to PCP decision. All patients had completed 12^th ^grade and had outpatient visit insurance coverage. All patients had well-controlled hypertension and diabetes, mild anemia (hemoglobin level of 11.5 mg/dL), and a GFR equal to 20 ml/min/1.73 m^2^, a level of renal function at which referral to the nephrologist is recommended [3].

### Covariates

Survey-based factors were assessed using a questionnaire previously tested in a pilot study, including previously-validated instruments [12]. Covariates included PCP demographic and practice information, disease-specific knowledge, attention to patient characteristics, experience with older CKD patients, expectations of the nephrology referral, risk aversion, and level of clinical uncertainty. PCPs' personal and practice demographic information included age, gender, race/ethnicity, foreign medical school graduate status, primary specialty, and community of practice (urban or non-urban). PCPs reported whether they were aware of existing KDOQI guidelines for management of CKD [3]. PCPs rated the importance of patient characteristics (patient age, race, medical co-morbidities, functional status, cognitive status, and the severity of CKD) in the decision to refer to a nephrologist. PCPs estimated the percentage of adults over age 65 in their practice, the number of referrals to nephrology they made in the last year, and the remaining life expectancy for each vignette patient. Potential PCP expectations of a nephrology referral were for: 1) blood pressure management; 2) treatment of anemia with erythropoietin: 3) discussion of CKD prognosis; 4) discussion about dialysis initiation; and 5) discussion of goals of care. PCP level of clinical uncertainty was evaluated using the validated Physician Reaction to Uncertainty Scale comprised of subscales for anxiety about uncertainty, concern about bad outcomes, reluctance to disclose uncertainty to patients, and reluctance to disclose uncertainty to other providers [19]. PCP level of risk aversion was assessed using a general question about risk taking behavior: "compared to other people your age, would you say you are": less willing to take risks, equally willing to take risks, or more willing to take risks.

### Outcomes

The primary outcome was whether the provider would recommend referral of the vignette patient to the nephrologist (yes or no).

### Participants

Participants were a purposeful sample of two community-based PCP provider groups who were active members of two Practice-based Regional Networks (PBRNs): the Upstate New York Network (UNYNET) based in Buffalo, New York and the Oklahoma Practice Research Network (OKPRN) based in Oklahoma City, Oklahoma. Network members were invited to participate in the survey because of their interest in CKD and because of their prior experience participating in online surveys [20]. The study was approved by the Institutional Review Boards of the Biological Sciences Division at the University of Chicago and the University of Oklahoma Health Sciences Center; and exempted at the State University of New York at Buffalo.

### Survey Administration and Recruitment

A total of 204 PCPs were invited to participate in the survey via email by the network director and provided informed consent. PCPs were sent follow-up emails every other week for 6 weeks. PCPs in the OKPRN who did not respond to email invitations after 6 weeks received a phone call and were mailed a written version of the survey. PCPs in the UNYNET who submitted the survey were given a $20 gift certificate to an online bookstore. PCPs in the OKPRN collectively chose to accept a payment of $1000 to the network general research fund rather than receive individual payments.

### Data Analysis

All analyses were conducted based on PCP responses to each vignette and accounted for clustering at the level of the provider. Bivariate analyses of patient and provider characteristics were evaluated by Spearman correlation, Wilcoxon-rank sum, chi-square or paired t-tests as appropriate. Sequential, random effects, multivariate logistic regression models were used to test the independent association of patient characteristics (Model 1), provider demographics (Model 2), and other provider characteristics including experience, expectations, disease-specific knowledge, risk aversion and uncertainty preferences (Model 3) with the decision to refer. Each model had new variables added as a block. All analyses were performed using STATA software version 11 (College Station, TX).

## Results

Eighty-five PCPs responded to the survey yielding a 42% response rate similar to that of other studies [2,9]. Respondents were overwhelmingly male (77.6%), white (90.2%), family practice physicians (89.3%), and the majority practiced in an urban setting (61.2%) (Table 2). The demographic characteristics of respondents were not statistically significantly different from those of non-responders (data not shown). The PBRNs were similar except in the report of awareness and use of KDOQI practice guidelines, which was significantly higher in the UNYNET group (100% versus 69.7%, p = 0.01). The vast majority of providers (87.1%) reported that the vignettes were "somewhat" or "very representative" of patients they see in their own practice.

**Table 2:** Demographics of primary care provider respondents

	Overall (n = 85)	UNYNET (n = 18)	OKPRN (n = 67)	P value
Age, mean (SD)	49.9 (9.7)	49.1 (8.9)	50.1 (9.8)	0.78
Female, (%)	22.4	27.8	20.9	0.53
Non-White, (%)	9.8	16.7	7.8	0.26
Family Practice Specialty, (%)	89.3	88.9	89.9	0.72
Urban Practice Setting, (%)	61.2	72.2	58.2	0.28
Estimated percentage of practice over age 65, mean (SD)	17(20)	3 (16.7)	14 (20.9)	0.25
Estimated number of patients referred to nephrology last year, mean (SD)	38 (44.7)	9 (50.1)	29 (43.2)	0.61
Self-reported awareness of Kidney Disease Outcomes Quality Initiative guidelines, (%)	76.2	100	69.7	0.01*
Vignettes "somewhat" or "very representative" of patients in practice, (%)	87.1	94.4	85.1	0.29

*Values reaching level of significance p < 0.05

Collectively, the respondent PCPs made 680 referral decisions. Despite all vignette patients being appropriate for referral based on KDOQI guideline recommendations, PCPs did not recommend referral in 257 (37.8%) of their decisions. PCPs most commonly cited multiple medical co-morbidities, no intervention warranted, and medical management that can be done in the PCP's office as reasons for non-referral.

The majority of PCPs reported that the severity of CKD was a "moderately" or "very important" factor in their decision to refer (69.5%). A much smaller group of providers noted that functional status (29.4%), cognitive status (32.9%) and the presence and severity of additional co-morbidities (45.9%) were at least "moderately" important in their referral decision. Only a few PCPs reported that patient age (16.5%) or race (3.5%) played an important role in their referral decision.

PCPs overwhelmingly expected nephrologists to evaluate the etiology of CKD (97%), discuss prognosis (98%), discuss the initiation of dialysis (94.1%), and discuss goals of care (96%) with referred patients. Most of them expected the nephrologist to manage CKD-associated anemia (64%), but not the blood pressure (40%).

### Bivariate Analyses

#### Patient factors

In bivariate analyses, older patient age (44.7% referred versus 58.8% non-referred, p < 0.001) and the presence of moderate dementia (41.4% versus 68.1%, p = 0.01) were strongly associated with a decision not to refer (Table 3). Other patient factors (and 2-factor interactions) were not statistically significantly associated with the referral decision including functional disability, as demonstrated by impaired ambulation requiring a walker (49.2% versus 51.4%, p = 0.58), and the presence of an additional serious co-morbidity, i.e., class III CHF, (50.1% versus 49.8%, p = 0.94).

**Table 3:** Bivariate analysis of referred versus not referred vignette patients (n = 680 decisions)

	Referred (n = 423)	Not Referred (n = 257)	P value
**Patient Characteristics**			

Older Age, (%)	44.7	58.8	< 0.001*

Female gender, (%)	50.4	49.2	0.81

Black race, (%)	51.3	47.9	0.38

Presence of stage III CHF, (%)	50.1	49.8	0.94

Ambulation with walker, (%)	49.2	51.4	0.58

Presence of moderate dementia, (%)	41.4	68.1	0.01*

**PCP Demographics**			

Older Age, mean (SD)	50.3 (9.89)	49.2 (9.26)	0.14

Non-white race, (%)	13.2	4.1	< 0.001*

Female gender, (%)	27.9	13.2	< 0.001*

OKPRN, (%)	75.4	84.4	0.01*

Urban practice, (%)	64.3	56.0	0.03*

Foreign Medical School Graduate, (%)	13.7	12.5	0.03*

**PCP Experience**			

Estimation of Remaining Life Expectancy, mean (SD)	6.11 (3.53)	5.38 (4.15)	0.02*

Report of knowledge of KDOQI guidelines, (%)	75.7	77.11	0.67

Importance of Patient Age, (%)	13.71	21.01	0.01*

Importance of Patient Race, (%)	4.73	1.56	0.03*

Importance of Co-morbidities, (%)	46.81	44.36	0.53

	28.13	31.52	0.35

Importance of Patient Cognitive Status, (%)	26.00	44.36	< 0.001*

Importance of Severity of CKD, (%)	76.60	57.59	< 0.001*

Estimated proportion of patients over age 65, mean (SD)	0.27 (0.44)	0.37 (0.48)	0.01*

More than 10 patients referred to nephrology in last 1 year, (%)	52.01	32.73	< 0.001*

**PCP Expectations**			

Expectation that nephrologist would evaluate etiology of CKD, (%)	63.00	37.00	0.01*

Expectation that nephrologist would manage blood pressure, (%)	63.29	36.67	0.65

Expectation that nephrologist would treat anemia, (%)	62.34	37.71	0.96

Expectation that nephrologist would discuss prognosis, (%)	62.19	37.83	0.98

Expectation that nephrologist would discuss initiation of dialysis, (%)	62.10	38.02	0.49

Expectation that nephrologist would discuss goals of care, (%)	63.40	36.60	0.001*

**PCP Reaction to Uncertainty and Risk Aversion**			

Anxiety about uncertainty^†^, mean (SD)	16.75 (4.20)	16.11 (3.78)	0.04*

Concern about bad outcomes^‡^, mean (SD)	8.84 (3.55)	8.40 (3.66)	0.12

Reluctance to disclose uncertainty to patients^§^, mean (SD)	17.47 (3.99)	17.85 (3.19)	0.20

Reluctance to disclose uncertainty to other providers^¢^, mean (SD)	4.54 (2.14)	4.45 (1.57)	0.54

Risk aversion, n (%)	77.8	68.1	0.01*

* Values reaching significance level p < 0.05; The Physician Reaction to Uncertainty Scale subscale ranges are ^†^6-30 points, ^‡ ^3-18 points, ^§ ^6-30 points, and ^¢ ^2-12 points

#### PCP factors

PCP mean estimated remaining life expectancy was significantly different between referred and non-referred patients (6.11 ± 3.53 versus 5.38 ± 4.15, p = 0.02). PCP demographic characteristics of female gender (27.9% versus 13.2%, p < 0.001) and non-white race (13.2% versus 4.1%, p < 0.001) were strongly associated with a decision to refer. PBRN affiliation (75.4% versus 84.4%, p < 0.001), urban practice setting (64.3% versus 56.0%, p = 0.03) and foreign medical school graduate status (13.7% versus 12.5%, p = 0.03) also were associated with the decision to refer (Table 3).

PCP rating of the importance of patient age (13.7 versus 21.0%, p = 0.01) and cognitive status (26.0 versus 44.3%, p = < 0.001) were associated with non-referral while rating the severity of CKD as more important was associated with referral (76.0 versus 57.6%, p < 0.001) (Table 3). PCP report of having referred more than 10 patients to nephrology in the previous year was associated with a decision to refer (52.0 versus 32.7%, p < 0.001) while having greater percentage of older patients in PCP practice (0.27 ± 0.44 versus 0.37 ± 0.48, p = 0.01) was associated with non-referral. PCP expectation of nephrologists to evaluate the etiology of CKD (63.0% versus 37.0%, p = 0.01) and discuss goals of care (63.4% versus 36.5%, p = 0.001) were associated with a decision to refer. PCP anxiety about uncertainty (16.75 ± 4.20 versus 16.11 ± 3.78, p = 0.04) and risk aversion (77.8% versus 68.1%, p = 0.01) were the only PCP attributes that were significantly associated with the decision to refer in the bivariate analysis.

### Multivariate logistic regression analyses

In random effects, multivariate logistic regression models, older patient age (OR = 0.27; 95%CI, 0.15 to 0.48) and the presence of dementia (OR = 0.14; 95%CI, 0.07 to 0.25) remained independently associated with lower likelihood of referral in the fully-adjusted model (Table 4). Longer estimated remaining life expectancy was not independently associated with the referral decision (OR = 0.99; 95%CI, 0.90 to 1.09).

**Table 4:** Multivariable analyses for the decision to refer vignette patients (n = 680 decisions)

	Model 1 OR (95% CI)	P value	Model 2 OR (95% CI)	P value	Model 3 OR (95% CI)	P value
**Patient Characteristic**						

Older Age	0.32 (0.20 to 0.51)	< 0.001*	0.28 (0.17 to 0.47)	< 0.001*	0.27 (0.15 to 0.48)	< 0.001*

Female gender	1.10 (0.70 to 1.72)	0.68	0.92 (0.57 to 1.46)	0.71	0.91 (0.55 to 1.50)	0.72

Black race	1.29 (0.83 to 2.03)	0.26	1.33 (0.83 to 2.12)	0.24	1.46 (0.89 to 2.41)	0.14

Presence of Stage III CHF	1.03 (0.66 to 1.61)	0.90	1.03 (0.65 to 1.64)	0.91	1.00 (0.60 to 1.68)	1.00

Ambulates with walker	0.83 (0.53 to 1.30)	0.42	0.85 (0.54 to1.36)	0.51	0.89 (0.54 to 1.46)	0.66

Presence of moderate dementia	0.16 (0.10 to 0.27)	< 0.001*	0.16 (0.09 to 0.27)	< 0.001*	0.14 (0.07 to 0.25)	< 0.001*

**PCP Demographics**						

Older Age			1.04 (1.01 to 1.06)	< 0.001	1.03 (0.97 to 1.09)	0.29

Non-white race			3.65 (1.67 to 7.95)	0.001*	30.29 (1.30 to 703.73)	0.03*

Female gender			3.42 (2.11 to 5.58)	< 0.001*	7.75 (2.07 to 28.93)	0.002*

OKPRN			0.60 (0.38 to 0.93)	0.02	0.33 (0.06 to 1.87)	0.21

Urban practice			1.10 (0.77 to 1.59)	0.60	0.64 (0.24 to 1.710	0.38

Foreign Medical School Graduate			1.09 (0.71 to 1.67)	0.69	1.80 (0.33 to 9.78)	0.50

**PCP Experience**						

Estimation of Remaining Life Expectancy					0.99 (0.90 to 1.09)	0.73

Report of knowledge of KDOQI guidelines					1.09 (0.30 to 3.98)	0.89

Importance of Patient Age					1.12 (0.20 to 6.23)	0.89

Importance of Patient Race					0.01 (0.00 to 0.31)	0.01

Importance of Patient Functional Status					1.28 (0.36 to 4.56)	0.70

Importance of Patient Cognitive Status					0.33 (0.08 to 1.25)	0.10

Importance of Severity of CKD					17.43 (4.90 to 61.93)	< 0.001*

Importance of Co-morbidities					0.22 (0.07 to 0.77)	0.02*

Majority of patients over age 65					1.07 (0.38 to 3.00)	0.89

More than 10 patients referred to nephrology in last 1 year					4.21 (1.54 to 11.48)	0.01*

**PCP Expectations of Referral**						

Expectation that nephrologist would evaluate etiology of CKD					2.16 (0.14 to 32.82)	0.58

Expectation that nephrologist would manage blood pressure					0.92 (0.33 to 2.58)	0.88

Expectation that nephrologist would treat anemia					0.28 (0.09 to 0.89)	0.03*

Expectation that nephrologist would discuss prognosis					0.20 (0.01 to 7.88)	0.39

Expectation that nephrologist would discuss initiation of dialysis					1.09 (0.10 to 12.40)	0.94

Expectation that nephrologist would discuss goals of care					53.13 (2.42 to 1168.00)	0.01*

**PCP Risk and Uncertainty**						

Anxiety about uncertainty					1.28 (1.04 to 1.57)	0.02*

Concern about bad outcomes					0.81 (0.67 to .97)	0.03*

Reluctance to disclose uncertainty to patients					0.92 (0.72 to 1.18)	0.50

Reluctance to disclose uncertainty to other providers					0.95 (0.73 to 1.25)	0.73

Risk aversion					3.39 (1.02 to 11.24)	0.05*

Sequential, random effects, multivariate logistic regression models were used to test the independent association of patient characteristics (**Model 1**), provider demographics (**Model 2**), and other provider characteristics including experience, expectations, disease-specific knowledge, risk aversion and uncertainty preferences (**Model 3**) with the decision to refer. Each model had new variables added as a block.

* Values significant to p < 0.05

Provider demographics, such as non-white race (OR = 30.29; 95%CI, 1.30 to 703.73) and female gender (OR = 7.75; 95%CI, 2.07 to 28.93), PCP report of the importance of CKD severity (OR = 17.43; 95%CI, 4.90 to 61.93) and greater numbers of referrals in the past year (OR = 4.21; 95%CI, 1.54 to 11.48) were associated with higher likelihood of referral. PCP expectation of nephrologist to discuss goals of care was also associated with higher likelihood of referral (OR = 53.13; 95%CI, 2.42 to 1168.00). PCP attributes including higher levels of anxiety about uncertainty (OR = 1.28; 95%CI, 1.04 to 1.57) and greater risk aversion (OR = 3.39; 95%CI, 1.02 to 11.24) were independently associated with the decision to refer. PCP concerns about bad outcomes was associated with lower likelihood of referral (OR = 0.81; 95%CI, 0.67 to 0.97).

## Discussion

CKD is an important health concern for older adults and yet they do not access nephrology services at the same rates as younger patients with equivalent severity of disease. In this study of PCP decision making about nephrology referral for hypothetical patients with severe CKD, we find that nearly 1/3 of older patients are not referred despite meeting guideline criteria for referral. We identified that older age and moderate dementia significantly reduce the likelihood that a patient will be referred while other patient factors including medical co-morbidity and functional disability do not impact such decisions. We also find that the likelihood of referral is impacted by provider characteristics and expectations independent of patient factors. These results suggest that referral decisions are complex in ways that current practice guidelines do not address.

Our finding that a large number of hypothetical patients with CKD are not referred to nephrology is consistent with previous studies of actual patient populations. Previous studies suggest that 33-85% of persons with moderate to severe CKD were unknown to nephrology services [5,7]. In several retrospective studies of CKD populations, older age is associated with lower likelihood of referral [5,7,9]. Our study further confirms that patient age is a strong predictor of non-referral.

However, our study also evaluated several other factors commonly associated with age to determine whether PCPs use age alone or as a proxy for other age-associated conditions. In our study, moderate dementia is independently associated with a lower likelihood of referral. Previous studies have not identified dementia as an independent factor for non-referral. Perhaps this is because dementia is under-recognized and under-diagnosed even in the CKD population where persons are at higher risk [21,22]. Contrary to other studies about CKD referral, medical co-morbidity is not associated with the referral decision in this study [9]. This finding may be a result of the study design in which we varied the presence or absence of a single co-morbidity (stage III CHF). The presence of stage III CHF alone may not influence the referral decision. Instead, provider referral decisions might be more strongly influenced by other significant co-morbidities [23]. Using this study design, we did not have the sample size to fully account for all the possible co-morbidities. Contrary to our previous findings, functional disability was not associated with the referral decision [12]. In these clinical scenarios, functional disability was represented as the use of an assistive device for ambulation. We hypothesized that PCPs would view the use of a walker as an indication of frailty and, therefore, they would be less likely to refer [12]. However, providers may not identify functional disability or frailty by the presence of impaired ambulation. Perhaps deficits in independent activities of daily living (IADLs), which we did not assess, are more important to providers. A fuller accounting of other relevant factors awaits future work.

PCP estimation of longer remaining life expectancy for a patient was significantly associated with the decision to refer in bivariate analyses although the difference was less than 1 year and of unclear clinical significance. However, estimated remaining life expectancy was not independently associated with likelihood of referral in multivariate analyses. Remaining life expectancy is a concept that incorporates age, gender, number/severity of medical co-morbidities, and the presence of functional disability into an estimation of physiologic age [24,25]. Arguably, remaining life expectancy could be used to evaluate patients instead of age given the significant heterogeneity of older adults where chronologic age may not be as important for outcomes [25]. In this study, PCPs exhibited significant variability in estimating remaining life expectancy suggesting that across PCPs there are inconsistencies in how providers calculate or conceptualize life expectancy. These inconsistencies in life expectancy calculations have important implications for the possible use of life expectancy rather than age in practice guidelines for CKD. If provider life expectancy estimates are significantly inaccurate, then the decisions made based on such estimates might be inappropriate. Future decision making studies could address how providers estimate life expectancy and determine whether providing an estimate of life expectancy would alter the referral decision.

In comparison to the few patient factors which affected the referral decision, we found several PCP factors that were statistically associated with the decision. Although several of these PCP factors have wide confidence intervals suggesting imprecise estimates due to the sample size, the potential implications of their influence on provider decision making should be further examined in future studies. The greater the importance of CKD severity to the PCP, the more likely he or she is to refer the patient, suggesting that raising PCP awareness about the health consequences of CKD might impact referral decisions. Also, PCPs who report referring greater numbers of patients to nephrology in the preceding year were more likely to refer the hypothetical patients. PCP expectation that the nephrologist would discuss goals of care, but not dialysis initiation, was very strongly associated with a higher likelihood of referral. Perhaps PCPs are reluctant or unprepared to discuss care goals with older patients who have severe CKD. This finding suggests that PCPs expect nephrologists to engage patients in a broader discussion about goals and not simply discuss dialysis. Interestingly, we find that PCP gender and race are independently associated with referral consistent with previous studies of referral behavior in other contexts [26-28]. It is unclear why this gender and race difference occurs. Our analyses are adjusted for PCP age, awareness of guidelines, reaction to uncertainty, and risk aversion, which might be hypothesized to vary by gender and race. While some of these factors exerted their own independent effects on the referral decision, they did not attenuate the effect of provider gender and race. Specifically, we find that PCP anxiety about clinical uncertainty, concern about bad outcomes, and greater risk aversion have significant independent effects on the referral decision. These results do suggest a potential role for these provider characteristics in decision making. The full meaning of the associations of these general measures of clinical uncertainty and risk aversion on referral decisions management of CKD awaits future work.

We hypothesize that PCP factors including expectations, personality traits, and risk preferences are important in decision making when the outcome of the decision is uncertain. In the case of referral to the nephrologist for severe CKD, PCPs confront several uncertainties including: 1) how likely is this older patient with CKD to progress to end-stage renal disease; 2) will the patient have strong preferences about starting dialysis if it is indicated; or 3) will the referral to nephrology result in improved outcomes for this individual patient. Given the lack of evidence to answer these uncertainties, it is likely that PCP characteristics, expectations, and personality traits will be more important in the decision making and contribute to practice variation. As evidence accumulates to address these uncertainties and guidelines incorporate this evidence into algorithms for risk assessment of older adults, the effects of these PCP characteristics on the referral decision may diminish. It remains to be seen if CKD-focused educational efforts targeting PCPs would eliminate the gender and race discrepancies or reduce the effect of personality traits in referral decision making.

There are several limitations to this study. First, this is a study of decision making using hypothetical vignettes. Decision making studies that involve clinical vignettes are highly correlated with decisions made during actual patient encounters; and vignettes are a cost-effective means of evaluating decisions that may not be easily studied in routine practice [29,30]. In this study, the careful control over variables of interest (e.g. GFR) provides insight into the specific role of the selected patient factors in the referral decision, but somewhat limits inferences for actual practice. Specifically, the effect of other medical co-morbidities associated with CKD, but not included in this study, on the referral decision was not assessed. Future studies involving actual patients would be needed to supplement this work and help to determine if these patient factors, or other disease-specific factors such as malnutrition or hypoalbuminemia, influence referral practices. Second, this is a study of a relatively small group of PCPs in practice-based research networks. As such, these results may not generalize to PCPs in other settings. However, this study did involve community-based PCPs from different types of practices and settings, in 2 different regions of the country. These findings are similar to results of our previous study of internists, geriatricians, and nephrologists at an academic institution [12,23]. The lower response in this study is comparable to other physician surveys and is typical of internet-based studies [2,9]. A larger, more representative sample of practicing providers would be needed to further test these findings. Third, through the use of the vignettes, we controlled for the degree of CKD and patient preferences, both of which may be important in the PCP's decision to refer the patient. Future studies could vary GFR to determine if providers use a threshold level of GFR to decide whether to refer regardless of other factors and whether this threshold differs from current guideline recommendations. We also recognize that patient (and family) preferences are important. The assumption that patients have no strong preferences about referral to a nephrologist may be incorrect. Perhaps patients (and families) equate a referral to nephrology with a decision about initiating dialysis. If this is true, then educating patients and PCPs about the important role of the nephrologist in managing severe CKD might improve referral rates. Additional studies are needed to understand patient expectations of a nephrology referral.

## Conclusions

Our findings provide new insight into PCP referral decisions for older adults with severe CKD to the nephrologist. In this study of PCP decision making using hypothetical clinical vignettes to systematically vary specific patient factors, we identify previously unrecognized patient (e.g. cognitive impairment) and provider (e.g. risk preferences) factors which independently predict which older patients with severe CKD are more likely to be referred by PCPs to a nephrologist. Additional work is necessary to determine if geriatric conditions like cognitive impairment and provider characteristics are also important in the outcomes of older patients with advanced CKD. Still, the results of this study underscore the complexity of the referral decision where a seemingly simple clinical recommendation--to refer an older adult with severe CKD as guidelines recommend--may involve challenging tradeoffs among multiple factors.

## Competing interests

The authors declare that they have no competing interests. Dr. Chester Fox is the director of the UNYNET practice-based research network. Dr. James W. Mold is the director of the OKPRN practice-based research network.

## Authors' contributions

KHC, SGS, JH, NS, MHC, and WD were involved in the study concept and design. KHC, SGS, CF, and JWM were involved in the acquisition of subjects for this study. KHC, JH, AMO, and WD were involved in the analysis and interpretation of the data. All authors made significant contributions to the preparation of the manuscript. All authors read and approved the final version of the manuscript.

## Pre-publication history

The pre-publication history for this paper can be accessed here:

http://www.biomedcentral.com/1471-2369/12/47/prepub
